# Development and validation of a novel prognostic model for patients with surgically resected esophageal squamous cell carcinoma

**DOI:** 10.3389/fonc.2022.955353

**Published:** 2022-08-18

**Authors:** Haiyang Hu, Jun Zhang, Hang Yan, Chao Qin, Haiyang Guo, Tao Liu, Shengjie Tang, Haining Zhou

**Affiliations:** ^1^ Department of Thoracic Surgery, Suining Central Hospital, An Affiliated Hospital of Chongqing Medical University, Suining, China; ^2^ Institute of Surgery, Graduate School, Zunyi Medical University, Zunyi, China; ^3^ Institute of Surgery, Graduate School, Chengdu University of TCM, Chengdu, China

**Keywords:** esophageal squamous cell carcinoma, prognostic model, nomogram, overall survival, following esophagectomy

## Abstract

**Background and objectives:**

Esophageal squamous cell carcinoma (ESCC) is the most common pathological type of esophageal malignancy in most regions of the world. The study aimed to identify risk factors and develop a predictive model for ESCC following surgical resection.

**Patients and methods:**

A total of 533 ESCC patients who underwent surgical resection from Suining Central Hospital were enrolled in the study. Cox proportional hazards regression and Least Absolute Shrinkage and Selection Operator (LASSO) regression were performed to identify significant prognostic factors. A prognostic model was constructed, and the receiver operating characteristic (ROC) curve, concordance index (C-index), and decision cure analysis (DCA) were used to evaluate the discrimination and calibration of the prognostic model. Subsequently, we built a nomogram for overall survival (OS) incorporating the prognostic factors, and a calibration plot was employed to assess the consistency between the predicted survival and the observed survival. Based on the model risk score, we split the patients into two subgroups, low-risk and high-risk, and we analyzed the survival time of these two groups using Kaplan–Meier (K-M) survival plots.

**Results:**

Five independent prognosis factors were identified as independent risk factors for OS in ESCC patients who underwent surgical resection. The C-index, ROC curve, and DCA showed that the prognostic model had good predictive accuracy and discriminatory power in the training cohort and validation cohort than other clinical features. A nomogram consisting of prognosis factors showed some superior net benefit. K-M survival plots showed significant differences in OS between the low-risk and high-risk groups. Similar results were observed in the subgroup analysis based on age, grade, and stage. Univariate and multivariate Cox regression analyses revealed that both risk score and risk group are independent prognostic factors in the patient cohort.

**Conclusions:**

This study put forward a novel prognostic model based on clinical features; biopsy data and blood biomarkers may represent a promising tool for estimating OS in ESCC patients.

## Introduction

Esophageal cancer (EC) is one of the most common and aggressive malignant tumors of the digestive system, and its incidence has been increasing in recent years (1). EC can be subdivided into esophageal squamous cell carcinoma (ESCC) and esophageal adenocarcinoma (EAC). The former is the most common pathologic type in EC hotspots worldwide, accounting for approximately 90% of all histological subtypes (2).

Esophagectomy, recommended as the preferred curative treatment as the mainstay of curative treatment for ESCC, is still considered a life-threatening gastrointestinal procedure with high mortality rates ranging from 8% to 23% (3, 4). Therefore, it is crucial to identify a series of new prognostic markers that can accurately predict the prognosis of the procedure and help to develop an individualized treatment plan in advance for ESCC patients intending to undergo esophagectomy.

The tumor–node–metastasis (TNM) staging system which was developed by the American Joint Committee on Cancer (AJCC) is widely used to predict the prognosis of cancer patients and guide treatment strategies (5). However, the TNM staging system mainly focuses on pathological outcomes but ignores other parameters of patients which may result in an insufficient accurate prediction of survival of esophageal cancer patients. Some scholars have suggested that including more clinical features in consideration could result in better prognostic accuracy and efficacy in several other cancer types (6–8). Therefore, it is necessary to establish a prediction model with additional prognostic factors for ESCC patients treated with esophagectomy for further study.

Studies published in recent years have revealed that inflammation and nutrition are considered as markers of tumor prognosis, which can be evaluated by hematological parameters, such as neutrophil-to-lymphocyte ratio (NLR), systemic inflammation score (SIS), platelet-to-lymphocyte ratio (PLR), lymphocyte-to-monocyte ratio (LMR), and prognostic nutritional index (PNI) (9–11). Currently, prognostic models for thoracic esophageal squamous cell carcinoma patients after radical esophagectomy based on blood biomarkers have rarely been developed.

In the current study, we identified prognostic factors from clinical characteristics, blood biomarkers, and tumor biopsy parameters based on the data of our institution. Furthermore, we developed a novel nomogram to predict the survival of patients with ESCC after surgical resection.

## Materials and methods

### Patient cohort and data collection

This study included patients treated with radical resection from January 2013 to December 2019, who were diagnosed with ESCC at the Thoracic Surgery Department of Suining Central Hospital. Data were randomly divided into training set and validation set by 7:3. Patients who met the following inclusion criteria were recruited for this study (1): Patients with histological diagnosis of resectable ESCC were included. Other histological types would be excluded (2). Esophagectomy *via* Ivor Lewis, Sweet, and McKeown procedures were included. Other procedures would be excluded (3). Patients with complete clinical information, blood biomarker indexes, follow-up data, and biopsy report (4). Patients were included without any other malignancies or distant metastases (5). The laboratory data were obtained within 7 days before surgery. The protocol of this research has been approved by the Clinical Research Ethics Committee of Suining Central Hospital, and informed consent has been exempted in the Ethical approval documents.

The following parameters were collected from each enrolled patient including baseline clinical information: gender, age, history of smoking, history of drinking, 8th AJCC TNM staging (12). Other clinical information included complications such as hypertension and diabetes. Blood biomarkers include albumin (ALB), hemoglobin (HGB), platelet (PLT), γ-glutamyl transpeptidase (γ-GT), alanine aminotransferase (ALT), aspartate aminotransferase (AST), serum iron (SI), cholesterol (CHOL), triglyceride (TG), creatinine (Cr), alkaline phosphatase (ALP), cystatin C (Cys-C), red blood cell distribution width (RDW), globulin (GLB), triglyceride glucose index (TyG) (13), albumin-to-globulin ratio (AGR), neutrophil-to-lymphocyte ratio (NLR) (14), derived neutrophil-lymphocyte ratio (dNLR) (15), lymphocyte-to-monocyte ratio (LMR) (16), platelet-to-lymphocyte ratio (PLR) (17), albumin-to-fibrinogen ratio (AFR) (18), prognostic nutritional index (PNI): albumin (g/L) + 5 × lymphocyte count × 10^9^/L (19), and systemic inflammation score (SIS), wherein one point is allocated for patients with ALB <40 g/l or LMR <4.44. The lowest score is 0, and the highest score is 2 (20). For the Naples prognostic score (NPS), one point is allocated for patients with CHOL <180 mg/dl, NLR >2.96, ALB <40 g/, or LMR <4.44. The lowest score is 0, and the highest score is 4 (21). Biopsy results included grade and log odds of positive lymph nodes (LODDS): lg[positive nodes + 0.5/(total nodes – positive nodes) + 0.5] (22). Baseline clinical parameters were not involved in feature screening for the terminal model.

### Follow-up investigation

Regular follow-up assessments began on the day of surgery. Patients were followed up every 3 months in the first 2 years, every 6 months for the next 3 years, and once a year after 5 years. The follow-up results were obtained from our medical records. The last follow-up for all patients was completed in December 2021. Follow-up assessments included routine laboratory tests, computed tomography (CT) scans of head and neck, chest and abdomen, and endoscopy when necessary.

### Statistical analyses

**Figure 1:**
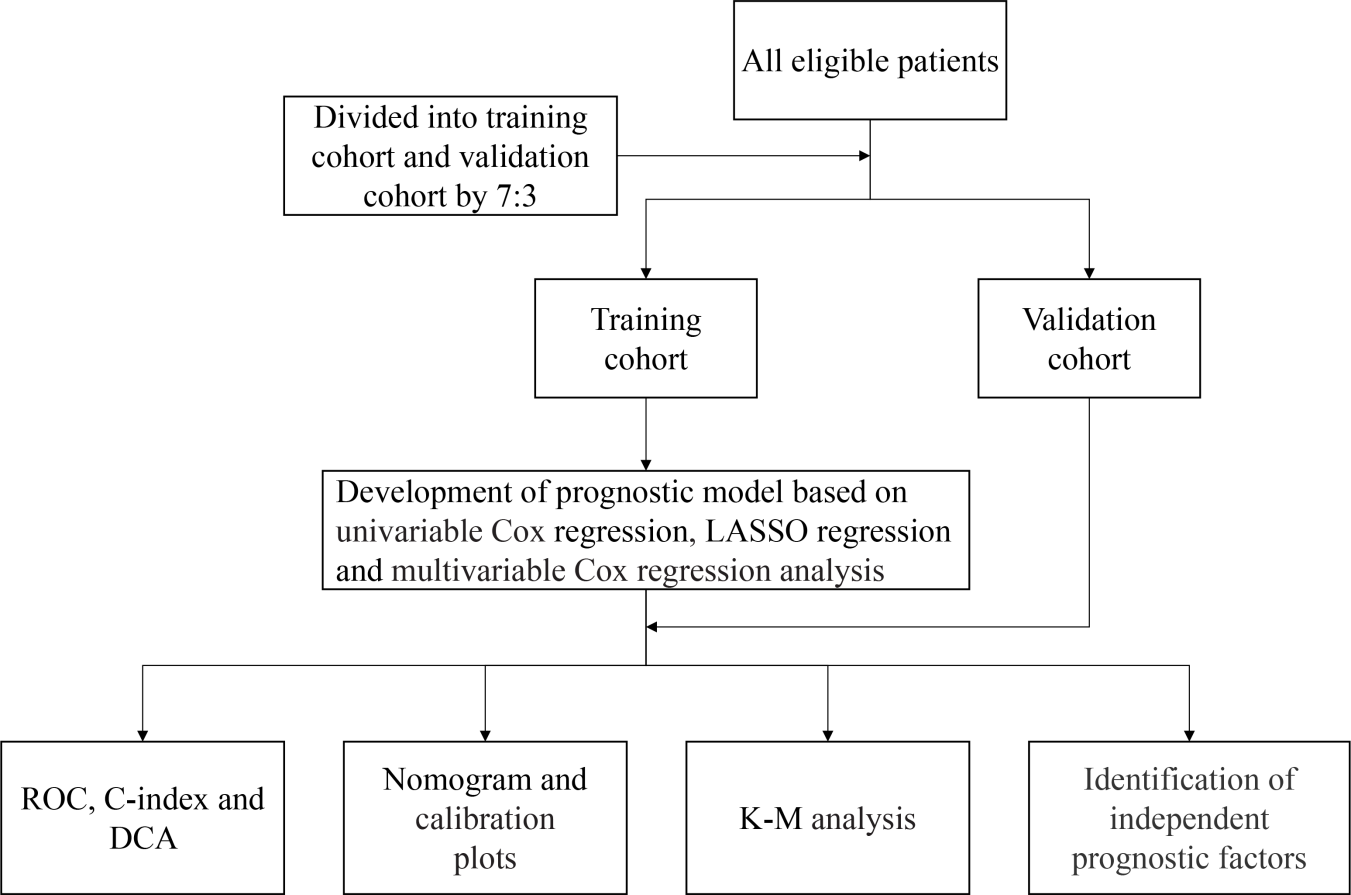
The flow diagram of the study.

Statistical analyses were performed using IBM SPSS 26.0 (version 26.0, SPSS Inc., Chicago, IL, USA) and R (version 4.1.1, the R Foundation for statistical computing). The chi-squared test was used to compare categorical variables, and the Mann–Whitney U test was used to compare the continuous variables. Univariate Cox regression with a threshold of *P*-value <0.05 was performed to screen variables related to patients’ prognosis. Then, Least Absolute Shrinkage and Selection Operator (LASSO) regression selects variables correlated with the measured outcome by shrinking coefficients’ weights down to zero for the ones not correlated with the OS in ESCC patients. To compare the predictive accuracy for individual survival between the prognostic model and other baseline clinical features, we evaluated the receiver operating characteristic (ROC) curve, concordance index (C-index), and decision curve analysis (DCA). Nomograms for the prediction of OS were built based on prognostic factors. The calibration plots of nomograms were used to assess the consistency between the predicted survival and the observed survival. Finally, the patients in the training and validation cohorts were split into low-risk and high-risk groups according to the median cutoff of the risk score. The Kaplan–Meier method and log-rank tests were used to assess differences in OS between the predicted high-risk and low-risk groups. Results with *P*-values of < 0.05 were considered statistically significant. **Figure 1** indicates the flow diagram of the study.

## Results

### Clinicopathological characteristics of patients

In this research, 373 eligible patients were enrolled in the training cohort, and 160 patients were included in the validation cohort. The median follow-up duration was 49.4 months for the training cohort and 52.0 months for the validation cohort. In the training cohort, the 1-, 3-, and 5-year OS rates were 95.2%, 71.4%, and 47.9%, respectively. In the validation cohort, the 1-, 3-, and 5-year OS rates were 95.6%, 73.8%, and 52.8%, respectively. Patients’ clinical features and laboratory indexes are listed in **Table 1**. There was no significant difference in the distribution of all parameters between the training cohort and validation cohort.

**Table 1:** Demographics and clinical characteristics of patients in the training and validation cohorts.

Variables	Training set n = 373	Validation set n = 160	*P*-value
Categorical variables (n%)			
Gender			0.691
Male	288 (77.2)	121 (75.6)	
Female	85 (22.8)	39 (24.4)	
Smoke			0.636
Ever	225 (60.3)	93 (58.1)	
Never	148 (39.7)	67 (41.9)	
Drink			0.673
Ever	218 (58.4)	93 (58.1)	
Never	155 (41.6)	67 (41.9)	
Hypertension			0.673
Presence	33 (8.8)	16 (10)	
Absence	340 (91.2)	144 (90)	
Diabetes			0.086
Presence	20 (5.4)	15 (9.4)	
Absence	353 (94.6)	145 (90.6)	
Grade			0.335
1	82 (22.0)	34 (21.3)	
2	219 (58.7)	103 (64.4)	
3	72 (19.3)	23 (14.4)	
T stage			0.323
1	54 (14.5)	28 (17.5)	
2	77 (20.6)	38 (23.8)	
3	212 (56.8)	87 (54.4)	
4	30 (8.0)	7 (4.4)	
N stage			0.519
0	195 (52.3)	77 (48.1)	
1	124 (33.2)	52 (32.5)	
2	44 (11.8)	26 (16.3)	
3	10 (2.7)	5 (3.1)	
AJCC stage			0.412
1	58 (15.5)	28 (17.5)	
2	145 (38.9)	55 (34.4)	
3	158 (42.4)	75 (46.9)	
4	12 (3.2)	2 (1.3)	
SIS			0.621
0	71 (19.0)	33 (20.6)	
1	192 (51.5)	75 (46.9)	
2	110 (29.5)	52 (32.5)	
NPS			0.138
0	38 (10.2)	16 (10)	
1	103 (27.6)	37 (23.1)	
2	114 (30.6)	53 (33.1)	
3	85 (22.8)	29 (18.1)	
4	33 (8.8)	25 (15.6)	
Continuous variables (mean ± SD)			
Age (years)	62.0 ± 7.6	61.1 ± 8.0	0.285
ALB (g/L)	41.0 ± 3.8	40.3 ± 4.3	0.057
HGB (g/L)	132.7 ± 16.0	132.0 ± 16.4	0.984
PLT (10^9/L)	185.8 ± 64.4	185.6 ± 60.9	0.708
γ-GT (U/L)	26.5 ± 29.1	27.1 ± 45.1	0.430
ALT (U/L)	20.2 ± 16.9	20.1 ± 12.1	0.435
AST (U/L)	23.2 ± 10.5	24.2 ± 9.9	0.088
SI (μmol/L)	15.2 ± 6.7	14.8 ± 7.2	0.301
CHOL (mg/dL)	179.5 ± 34.6	173.3 ± 36.6	0.063
TG (mg/dl)	100.4 ± 57.2	96.2 ± 46.3	0.473
Cr (μmol/L)	74.7 ± 16.8	73.0 ± 15.7	0.278
ALP (U/L)	73.7 ± 21.1	77.1 ± 38.0	0.561
Cys-C (mg/L)	0.9 ± 0.2	0.9 ± 0.2	0.061
RDW (fL)	45.6 ± 4.7	45.4 ± 4.9	0.231
GLB (g/L)	29.6 ± 4.6	29.1 ± 4.2	0.612
TyG	8.3 ± 0.5	8.2 ± 0.5	0.211
AGR	1.4 ± 0.2	1.4 ± 0.2	0.325
NLR	2.74 ± 1.9	3.5 ± 4.9	0.328
dNLR	1.8 ± 1.1	2.1 ± 1.5	0.150
LMR	3.8 ± 1.7	3.8 ± 1.7	0.508
PLR	128.3 ± 56.3	135.9 ± 84.9	0.791
LODDS	-0.9 ± 0.5	-0.9 ± 0.5	0.353
PNI	48.9 ± 5.0	48.3 ± 5.9	0.563
AFR	11.5 ± 2.8	11.5 ± 3.0	0.633

### Construction of the novel prognostic model

**Figure 2:**
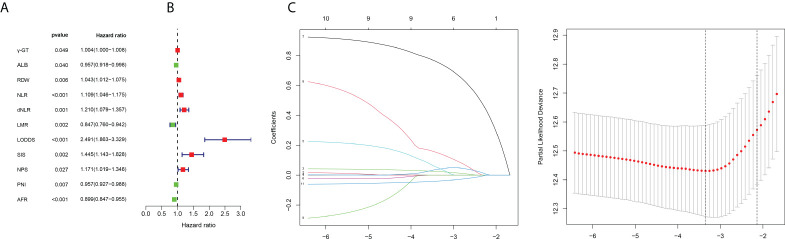
Filtering of variables. **(A)** Forest map of 11 prognosis-related variables based on univariate Cox regression. **(B)** LASSO coefficient profiles of candidate variables. **(C)** Ten-fold cross-validation results that identified optimal values of the penalty parameter λ.

Eleven OS-related variables were identified by univariate Cox regression analysis (**Figure 2A**). LASSO regression was employed to reduce the overfitting variables (**Figures 2B, C**). Then, multivariate Cox regression analysis was performed to construct a prognostic model composed of five variables: RDW, dNLR, LODDS, SIS, and AFR. Then, the prognostic model risk score for each patient was computed according to the summation of five variables multiplied by their coefficient: *risk score* = *RDW* × 0.04077 + *dNLR*× 0.16583 + *LODDS*× 0.89097 + *SIS* × 0.25125−*AFR*× 0.05711.

### The predictive accuracy of the new prognostic model compared with other clinical features

**Figure 3:**
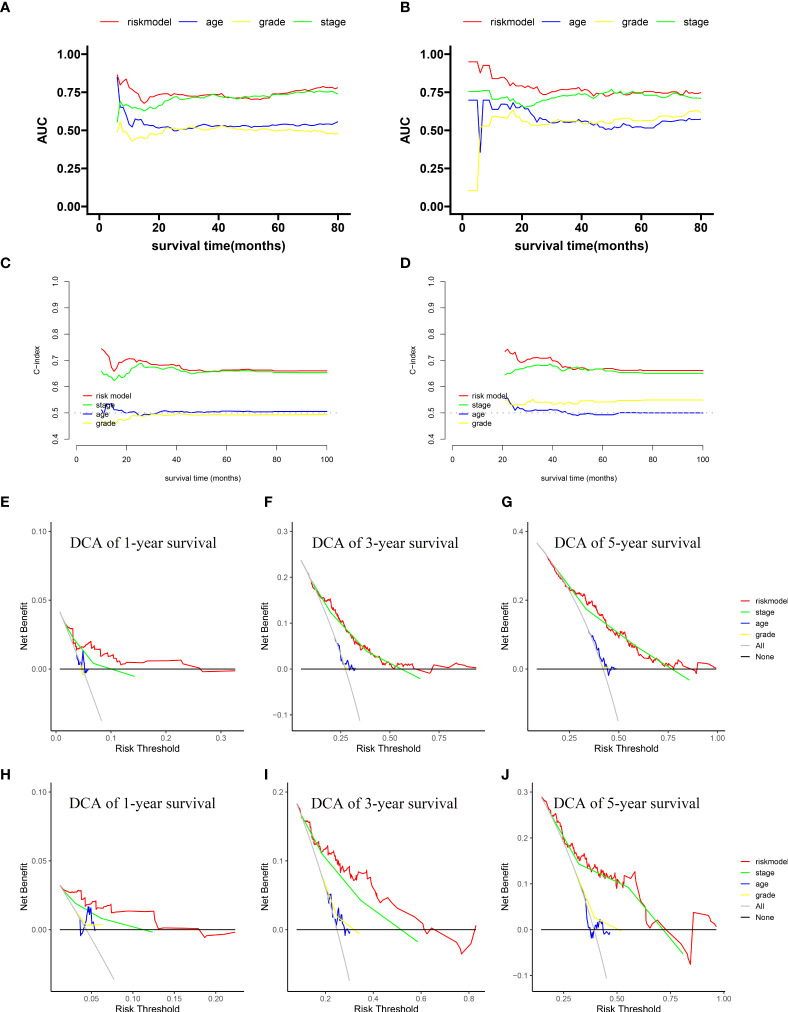
Comparison of the predictive accuracy and discrimination of the novel prognostic model with other clinical features. Time-dependent ROC in the training cohort **(A)** and validation cohort **(B)**. Continuous C-index curves in the training cohort **(C)** and validation cohort **(D)**. One-, 3-, and 5-year DCA plots in the training cohort **(E–G)** and validation cohort **(H–J)**.

We compared the area under the ROC curve (AUC) between the novel prognostic model, age, grade, and stage using time-dependent ROC. In most of survival time, the AUC of our novel prognostic model was higher than the others, in both the training cohort **(** **Figure 3A** **)** and the validation cohort **(** **Figure 3B** **)**. The continuous C-index curve indicates that the prognostic model has better discrimination ability than the TNM staging system **(** **Figures 3C, D** **)**. The 1-, 3-, and 5-year DCA showed that the prognostic model had a better overall net benefit than that of the TNM staging system, grade, gender, and age across a wide range of reasonable threshold probabilities in the training cohort and the validation cohort **(** **Figures 3E–J** **)**. These results indicated that the novel prognostic model displayed better accuracy in predicting OS compared with other clinical features.

### Building and validating a predictive nomogram

**Figure 4:**
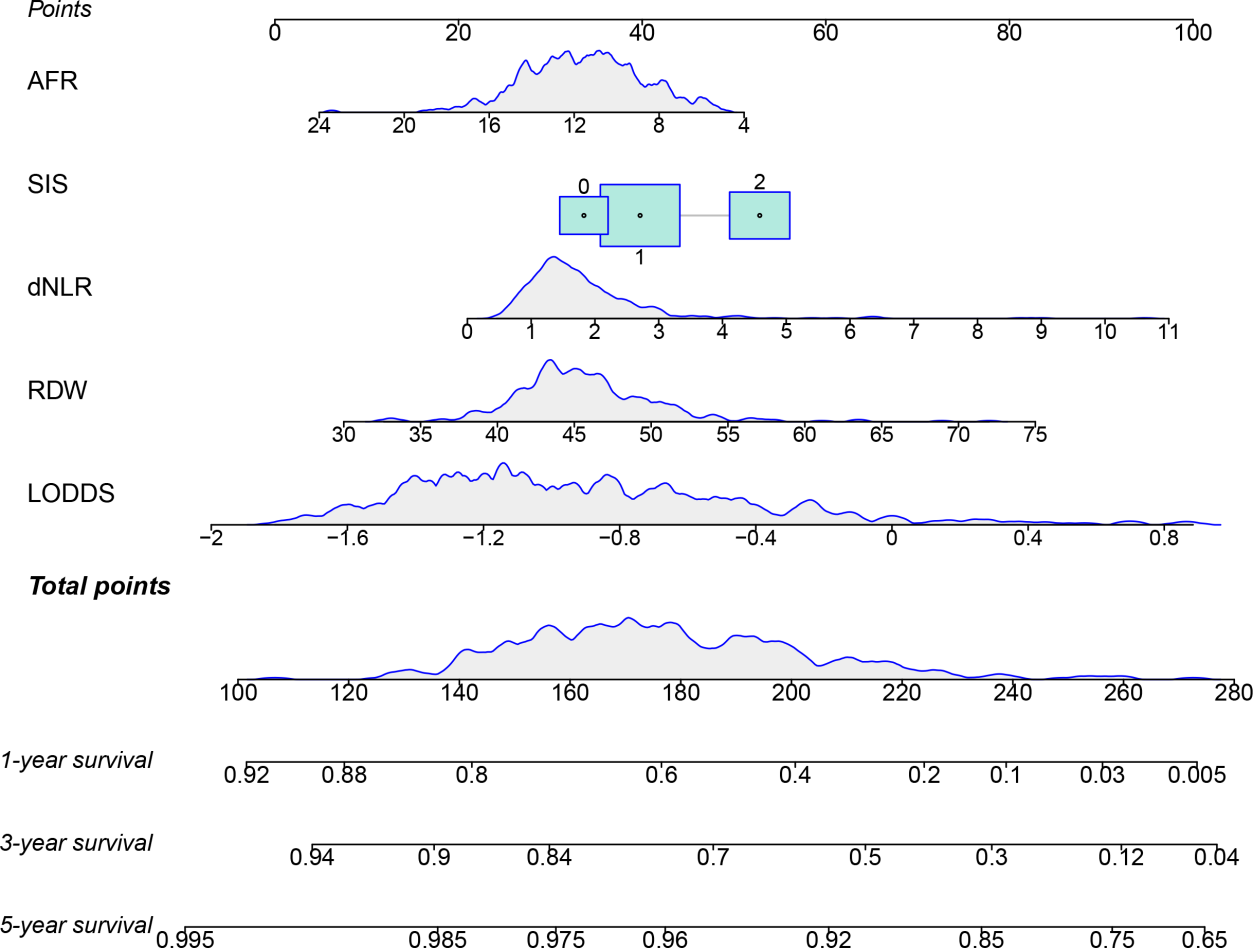
The nomogram for predicting the 1-, 3-, and 5-year survival of ESCC patients after radical esophagectomy.

**Figure 5:**
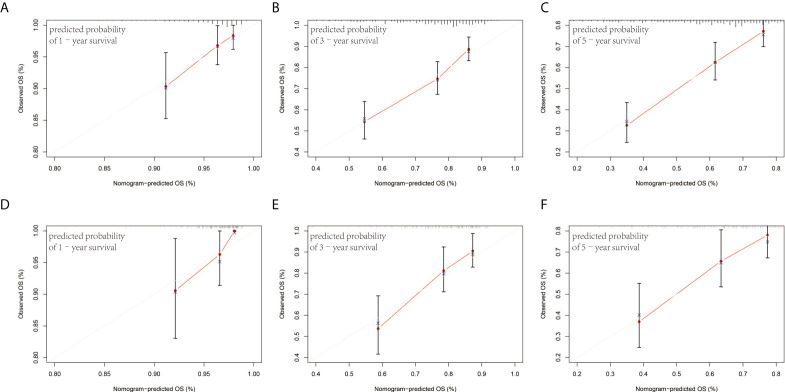
Calibration plots of the nomogram for 1-, 3-, and 5-year survival prediction in the training cohort **(A–C)** and validation cohort **(D–F)**.

The prognostic factors of the model were integrated into a nomogram to predict the 1-, 3-, and 5-year OS in the training cohort **(** **Figure 4** **)**. Calibration curves for the nomogram revealed no deviations from the reference line, demonstrating a good match between the probabilities predicted and the actual observations **(** **Figures 5A–F** **)**.

### Survival analyses of ESCC patients according to prognostic model risk score

**Figure 6:**
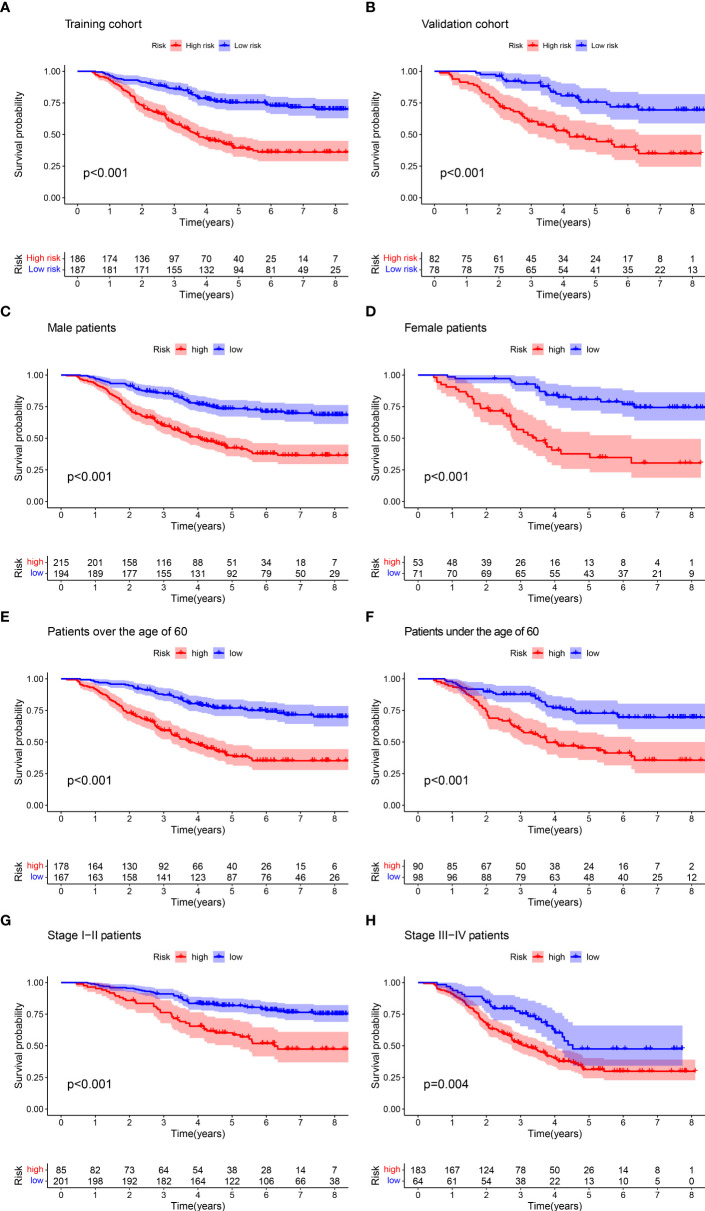
Kaplan–Meier curves for the OS of patients in the high- and low-risk groups. There were significant differences in the survival of high- and low-risk patients in the training group **(A)** and the validation group **(B)**, the male group **(C)** and the female group **(D)**, the older group **(E)** and the younger group **(F)**, and the early group **(G)** and the advanced group **(H)**.

We classified patients into two different subgroups based on the median cutoff of the training cohort: low-risk group (risk score ≤0.933) and high-risk group (risk score >0.933). Kaplan–Meier curves were compared to assess the differences in survival between low-risk and high-risk groups. The low-risk group showed a significantly longer OS than the high-risk group for both cohorts **(** **Figures 6A, B** **)**. In addition, we performed subgroup analysis for all patients according to age (≤60, >60), gender, and stage (I–II, III–IV), respectively. The results revealed a significant difference between low-risk and high-risk groups in different ages, genders, and stages **(** **Figures 6C–H** **)**. Finally, risk score, risk group, and other variables were included in univariate and multivariate Cox regression analyses. As shown in **Table 2**, both risk score and risk group are independent prognostic factors in the patient cohort.

**Table 2:** Univariate and multivariate Cox regression analyses for survival.

Variables	Univariate Cox regression analysis			Multivariate Cox regression analysis		
	HR	95% CI	*P*-value	HR	95% CI	*P*-value
Age	1.012	0.994-1.029	0.188	1.012	0.994-1.030	0.190
Gender (male~female)	0.847	0.615-1.167	0.310			
Smoke (never~ever)	1.193	0.908-1.568	0.205			
Drink (never~ever)	1.477	1.120-1.947	0.006	1.260	0.949-1.672	0.109
Hypertension (absence~presence)	1.044	0.666-1.637	0.851			
Diabetes (absence~presence)	1.117	0.592-2.106	0.733			
T stage (1-4)	1.713	1.428-2.055	<0.001	1.342	1.078-1.670	0.008
N stage (0-3)	1.655	1.437-1.906	<0.001	0.918	.0705-1.197	0.529
AJCC stage (1-4)	2.158	1.791-2.601	<0.001	1.510	1.064-2.142	0.021
Grade (1-3)	1.041	0.844-1.284	0.709			
Risk (low~high)	3.136	2.354-4.178	<0.001	1.912	1.366-2.678	<0.001
Risk score	1.438	1.334-1.550	<0.001	1.219	1.096-1.357	<0.001

## Discussion

In the current study, we screened out five variables (RDW, dNLR, LODDS, SIS, and AFR) from clinical features, blood biomarkers, and biopsy parameters based on Cox regression and LASSO regression affecting the prognosis of ESCC patients. RDW is a parameter in red blood cell size variability and is used for estimating the pathogenesis of anemia (23). A growing body of evidence has suggested that high RDW is frequently influenced by inflammation and oxidative stress in predicting an increased overall and disease-specific mortality across patients with chronic or progressive inflammation diseases (24, 25). Warwick et al. have shown that preoperative RDW could predict OS of patients undergoing pulmonary resections for non-small cell lung cancer (26). Yoshida et al. suggested that preoperative RDW may be a predictor of postoperative pneumonia, postoperative severe morbidity, and reoperation in EC patients after open esophagectomy (27). The dNLR is calculated as the ratio of neutrophils to the difference between total leukocytes and neutrophils in peripheral blood (28). Its role to discriminate prognosis of cancer has been widely explored. Cox et al. demonstrated that an elevated preoperative dNLR is a potential independent prognostic marker for both progression-free survival (PFS) and OS in EC treated with chemoradiotherapy (29). Li et al. found that the dNLR was significantly associated with pathology grade, original tumor site, LDH, neutrophil count, lymphocyte count, and disease control rate in metastatic non-colorectal gastrointestinal cancer patients treated with immune checkpoint blockade and a higher level of dNLR was associated with shorter OS (30). LODDS, defined as the logarithm of the ratio between the number of positive lymph nodes and number of negative lymph nodes, has been proven to be a predictive power of prognosis in various cancers. The prognostic role of LODDS and the superiority of LODDS in predicting survival compared with either the traditional N stage or the lymph node ratio was confirmed in ESCC patients undergoing surgical resection. LODDS can serve as a candidate indicator to provide prognostic guidance for ESCC patients. Research by Patel et al. indicates that LODDS is an independent predictor of OS in the squamous cell carcinoma of the penis. It has a superior prognostic significance than lymph node density classification and AJCC N stage systems (31). Similarly, Yang et al. found that the LODDS stage demonstrated better prognostic performance than the AJCC N or lymph node ratio stage in ESCC patients after esophagectomy. It can be applied to evaluate the lymph node status to increase the precision of staging and evaluation of survival (22). SIS is established based on the combination of the pretreatment serum ALB and LMR. Measurements of the SIS are economical and timesaving in clinical practice. Since Chang et al. first reported that SIS predicts the postoperative prognosis of patients with clear-cell renal cell carcinoma (32), increasing studies have found the prognostic value of SIS in postoperative cancer patients. The retrospective study of Fu et al. found that SIS is an independent risk factor for ESCC patients undergoing radical esophagectomy and three-field lymphadenectomy, and addition of SIS to their multivariate model increases the predictive accuracy of the OS (20). Furthermore, higher SIS was associated with poorer OS in colorectal cancer, gastric cancer, etc. (33–35). AFR, the ratio of Alb to fibrinogen, combines these two biomarkers and amplifies the sensitivity for evaluating inflammation and nutrition status which has been widely recommended as a prognostic factor in various malignance tumors, such as operable non-small-cell lung cancer and operable soft-tissue sarcoma (36, 37). A retrospective study involving 365 elderly patients with gastric cancer suggested that the preoperative AFR level is a useful factor in predicting postoperative complications after radical laparoscopic gastrectomy (38). Chen et al. suggested that preoperative AFR can be an independent prognostic factor for non-small cell lung cancer patients, and a higher AFR can increase OS and DFS (39).

Based on the five factors above, we successfully developed a prognostic model to estimate the probability of OS for patients with ESCC who received radical esophagectomy. We compared the predictive accuracy and discrimination of the novel prognostic model with 8th TNM staging, age, and grade. In general, the prognostic model had good predictive accuracy and discriminatory power than others in both the training cohort and validation cohort. Finally, according to the risk score, we split the patients into high-risk and low-risk groups. There were significant differences in OS between the two groups of patients in both training cohort and validation cohort. Moreover, three types of subgroup analyses based on age, sex, and stage revealed similar results. These results indicated that the novel prognostic model had good predictive accuracy and discrimination for estimating OS for patients with ESCC who received radical esophagectomy, and it serves as a readily available tool for risk-stratifying patients and might be used as a stratification factor in future clinical trials aiming to optimize the treatment strategies for resectable ESCC patients.

However, there are limitations in our study. Our study is a retrospective study in one single center. More medical centers and samples could be added to optimize our evaluation system and solve the limitation. In conclusion, the prognostic model is a reliable tool for clinical decision making, but further validation is required to determine whether it could be applied to broader populations.

## Data availability statement

The original contributions presented in the study are included in the article/**Supplementary Material**. Further inquiries can be directed to the corresponding author.

## Ethics statement

This study was reviewed and approved by Ethics Committee of Suining Center Hospital; Suining Central Hospital. Written informed consent for participation was not required for this study in accordance with the national legislation and the institutional requirements.

## Author contributions

HH and HZ conceptualized the study. HH, JZ, and ST contributed to the methodology. HY and CQ conducted the formal analysis and investigation. HH, ST, and HG wrote and prepared the original draft. ST and HZ provided the resources and supervised the study. All authors contributed to the article and approved the submitted version.

## Conflict of interest

The authors declare that the research was conducted in the absence of any commercial or financial relationships that could be construed as a potential conflict of interest.

## Publisher’s note

All claims expressed in this article are solely those of the authors and do not necessarily represent those of their affiliated organizations, or those of the publisher, the editors and the reviewers. Any product that may be evaluated in this article, or claim that may be made by its manufacturer, is not guaranteed or endorsed by the publisher.
